# The Role of Cardiac Imaging in the Evaluation of Cardiac Involvement in Systemic Diseases

**DOI:** 10.7759/cureus.20708

**Published:** 2021-12-26

**Authors:** Kelash Kumar, Karthik Seetharam, Fnu Poonam, Amit Gulati, Adnan Sadiq, Vijay Shetty

**Affiliations:** 1 Department of Internal Medicine, Maimonides Medical Center, Brooklyn, USA; 2 Department of Cardiology, Wyckoff Heights Medical Center, Brooklyn, USA; 3 Department of Internal Medicine, Wyckoff Heights Medical Center, Brooklyn, USA; 4 Department of Cardiology, Maimonides Medical Center, Brooklyn, USA; 5 Department of Internal Medicine/Cardiology, Maimonides Medical Center, Brooklyn, USA

**Keywords:** cmr, echocardiography, systemic diseases, inflammatory cardiomyopathy, multimodality cardiac imaging

## Abstract

For systemic diseases like rheumatoid arthritis, systemic lupus erythematosus (SLE), systemic sclerosis, systemic vasculitis, myopathies, and mixed connective tissue diseases, cardiac disease is a major contributing factor for morbidity and mortality. The cardiovascular manifestations are the result of various pathophysiological components, which complicate management. Furthermore, the signs and symptoms can be subtle and missed due to the complex nature of the underlying condition. As a result, various imaging approaches play an imperative role in diagnosis and prognosis. The evolving role of these modalities could lead to risk stratification and improved therapies in the future. In conclusion, our review article will highlight the role of cardiac imaging in the evaluation of cardiac involvement for systemic diseases.

## Introduction and background

Systemic diseases encompass a broad spectrum of diverse pathologies with many overlapping clinical features like rheumatoid arthritis, systemic lupus erythematosus (SLE), systemic sclerosis, systemic vasculitis, myopathies, and mixed connective tissue diseases. Despite their heterogeneous nature, these disorders are linked by their cardiovascular involvement, which can affect diagnostic pathways [1]. Cardiac involvement in systemic diseases is associated with morbidity and mortality [1]. Despite the elevated mortality, cardiovascular disease (CVD) is underestimated due to its atypical and subtle presentations [2]. Although significant progress has been made in these fields, there still remain significant gaps in cardiovascular specific mechanisms in systemic diseases [1]. Furthermore, cardiac involvement can complicate treatment because of its accompanying complexity [3-4]. Nevertheless, various imaging modalities play a paramount role in navigating the next step in medical management [3]. The optimal diagnostic algorithm for each of these conditions is not clearly known and needs to be tailored according to the underlying condition [5]. In this review article, we explore the role of cardiac imaging in the evaluation of cardiac involvement in systemic diseases. 

The manifestations of CVD in systemic diseases can be attributed to a variety of pathophysiologic phenomena such as vascular inflammation, micro or macro ischemia, and myocardial fibrosis [2]. With the advent of new targeted therapy in these conditions, there has been a significant reduction in mortality [1]. Nevertheless, the life expectancy of these patients is comparatively lower than that of the general population [6]. CVD events have been associated with excess mortality risk in later stages of life. In addition, any part of the cardiac tissue can be affected leading to myocardial or pericardial inflammation, heart failure, and pulmonary artery hypertension (PAH) [4]. As aforementioned earlier, clinical presentation of CVD can be subtle and under-estimated in systemic diseases [4]. It must be emphasized that the presence of overt cardiac signs is suggestive of advanced disease, which is linked to ominous prognosis [7]. 

## Review

Role of imaging in systemic disease

The complex nature of CVD in systemic diseases compels the need for proper diagnostic modalities for adequately assessing the pathophysiological processes. Echocardiography, nuclear imaging, and x-ray coronary angiography have been long-established pillars in cardiovascular imaging (Table 1) [1]. The Achilles heel of these modalities is the inability to recognize the development of inflammation and fibrosis in the earlier stages (Table 2) [1]. As a result, their performance can be suboptimal. The emergence of cardiac magnetic resonance (CMR) has greatly shifted the paradigm in these diseases. CMR can be used to detect and monitor tissue characterization and cardiovascular pathogenesis in systemic diseases [1]. Recent developments in position emission tomography (PET)/computed tomography (CT) and PET/CMR have been considered to play a valuable role in these disorders. 

**Table 1:** Comparison of Different Imaging Approaches for Systemic Diseases

Noninvasive Imaging Techniques	Function	Cost	Radiation	Expertise	Spatial resolution	Strengths
Echocardiogram	Evaluates valves, pericardium, ventricular function	Low	No	No	None	Widely available, can be done at bedside
Single Photon Emission Computed Tomography	Evaluates myocardial function and ischemia	High	Yes	Yes	Low	Reasonable sensitivity, not very specific
Positron Emission Tomography	Evaluates myocardial function and ischemia	High	Yes	Yes	Low	Very sensitive and specific
Computed Tomography	Evaluates coronary arteries and great vessels	High	Yes	Yes	None	Widely available
Cardiac Magnetic Resonance Imaging	Evaluates tissue characterization, inflammation, perfusion, fibrosis	High	No	No	High	Highly reproducible, operator independent

**Table 2:** Comparison of Tissue Characterization Properties of Various Modalities in Systemic Diseases

Noninvasive Imaging Techniques	Inflammation	Ischemia	Scar	Vasculitis	Coronary Arteries	Microvascular dysfunction
Echocardiogram	No	Yes/ No	Yes/ No	Yes/ No	No	Yes/ No
Positron Emission Tomography and Single Photon Emission Computed Tomography	No	Yes/ No	Yes/ No	Yes/ No	No	No
Computed Tomography	No	No	Yes/ No	No	Yes	No
Cardiac Magnetic Resonance Imaging	Yes	Yes	Yes	Yes	Yes/ No	Yes

A Brief Overview Of Diagnostic Modalities In Systemic Diseases

Echocardiography is the first-line diagnostic modality used in a variety of conditions [1]. It can detect valvular changes during stress and rest. It must be noted that image quality is strongly dependent on the expertise of the operator and the acoustic window of the patient. As stated earlier, echocardiographic indices cannot measure cardiac tissue characterization [1]. Tissue Doppler imaging permits the evaluation of systolic and diastolic velocities. Additionally, it can measure myocardial deformation. It is hindered by angle dependency because deformation occurs in a two-dimensional plane, whereas the deformation of the myocardium occurs in three dimensions. Speckle tracking is not angle-dependent and can measure strain in the longitudinal, radial, and circumferential axis [4]. Unfortunately, it cannot detect the presence of edema or fibrosis, which are quintessential for risk stratification in CVD of systemic diseases [2].

In nuclear imaging, single-photon emission computed tomography (SPECT) and PET are frequently used in clinical cardiology. Nevertheless, these modalities have relatively low specificity for the identification of coronary artery disease (CAD). Similar to echocardiography, they cannot characterize tissue properties in systemic diseases [1]. There are a number of disadvantages, which include ionizing radiation, production of radiotracers, low spatial resolution [1-2].

Multi-slice CT can assess the calcification and extent of atherosclerosis in the coronary artery. It can evaluate for myocardial scarring. Very few studies have explored the possibilities of tissue characterization with CT. Some disadvantages include the high utilization of iodinated contrast agents and varying levels of expertise that have been limiting its growth in CVD of systemic diseases [1].

CMR is a non-invasive modality that utilizes photons for the production of various images. CMR enables excellent spatial resolution and tissue characterization. It is reproducible and provides valuable information regarding myocardial inflammation. A number of studies have explored the role of CMR in various systemic diseases (Table 3). Furthermore, it can provide valuable information even in the context of previous normal evaluation [5]. There is an absence of ionizing radiation, which allows repeat scans. Silent myocardial ischemia, fibrosis, peri-myocardial inflammation, CAD, vascular inflammation, and pulmonary hypertension can be assessed. Moreover, it can assess occult lesions such as myocarditis or edema. The most commonly used parameters in CMR include longitudinal relaxation time (T1) and transverse relaxation time (T2). T1 mapping, T2 mapping, and extra cellular volume (ECV) enable rapid tissue characterization and assessment of myocardial fibrosis.

**Table 3:** Table 3: Various Studies Using CMR in Systemic Diseases RA: rheumatoid arthritis; ECV: extracellular volume; CMR: cardiovascular magnetic resonance imaging; SLE: systemic lupus erythematosus; CAD: coronary artery disease

Study	Year	Disease	Number of subjects	Number of subjects
Greulich et al. [12]	2017	RA	22 RA; 20 C	RA patients had elevated T1, ECV, and T2 values
Ntusi et al. [13]	2015	RA	39 RA; 39 C	Focal and diffuse fibrosis in the myocardium was linked to abnormal strain and RA disease
Kobayashi et al. [32]	2014	RA	20 RA; 20 C	CMR was sued to assess the effect of tocilizumab in RA patients with left ventricular dysfunction
Puntmann et al. [48]	2013	SLE	33 SLE; 21 C	CMR detected various imaging patterns in SLE heart failure
Mavrogeni et al. [50]	2016	SLE	50 SLE	CMR identified abnormal findings in patients with normal non-invasive testing
Mavrogeni et al. [61]	2012	SSC	7 SSC; 12 C	Asymptomatic perfusion defects have been detected by adenosine stress CMR
Mavrogeni et al. [62]	2016	SSC	105 SSC	CMR revealed evidence of Q waves with myocardial fibrosis associated with systemic disease, not CAD
Murtagh et al. [70]	2016	Sarcoidosis	205	Scars detected by CMR was a predictor of arrhythmia and cardiovascular death
Crouser et al. [81]	2016	Sarcoidosis	8	T2 mapping can be used to monitor immunosuppressive treatment in cardiac sarcoidosis
Mavrogeni et al. [87]	2004	Vasculitis	13	CMR coronary angiography was equal to x-ray coronary angiography for coronary artery evaluation in Kawasaki disease

Limitations of CMR

There are still a number of limitations to CMR. Though late gadolinium enhancement (LGE) can be a useful approach, it cannot detect diffuse fibrosis [8]. LGE is not particularly useful for detecting diffuse fibrosis [9]. It can primarily identify areas of local fibrosis. It requires normal myocardium to serve as a reference point to distinguish areas of fibrosis. This can be viewed as a major limiting factor in LGE [9]. This issue can be circumvented by T1 mapping. However, T1 mapping is still a newer approach and is associated with technical issues related to cardiac and respiratory motion [10]. It cannot be utilized for patients with significant renal impairment [10]. The accuracy of T1 is reduced at longer T1 values. There are a number of mapping sequences with potential advantages and disadvantages. For example, the Look-Locker and rapid T1 mapping sequence do not have automatic features for heart rate correction [10]. Although ECV can measure interstitial space, it can be problematic in certain conditions [11]. There can be a potential overlap between normal and diseased myocardium [11]. There is still a debate regarding the effect of magnetic field ranges from 1.5 vs 3 T, on myocardial ECV [11]. T2 mapping is still a newer approach that requires further investigation [9]. CMR is contraindicated in patients with permanent pacemakers, automated implantable cardiac defibrillators, or patients with clips for cerebrovascular aneurysms [5]. It is not recommended during the first trimester of pregnancy [5]. 

Rheumatoid arthritis

Rheumatoid arthritis (RA) is a chronic inflammatory disorder involving a number of joints [4,12-13], greatly affecting the quality of life and contributing to a high socioeconomic burden [14]. The major contributing cause of mortality in RA is CVD [15], there is a high incidence of congestive heart failure in these patients [16]. There is particularly high demand for multi-modality imaging and especially CMR in identifying high-risk patients for early management [4].

Imaging Modalities in Rheumatoid Arthritis

Echocardiography is the fundamental cornerstone of cardiovascular examination and it is the most widely used noninvasive imaging technique [2]. Echocardiography can identify morphological, functional, and valvular disturbances during rest and stress [2]. Echocardiographic parameters in transthoracic echocardiography (TTE) such as elevated left atrial volume, abnormal E/A ratio, increased right atrial size have been seen in previous reports comparing RA to healthy individuals [17-18]. Impaired diastolic pressure has been associated with extra-articular manifestations [19]. In one report, RA patients have had impaired diastolic function in relation to controls on tissue Doppler imaging [20]. Transesophageal echocardiography (TEE) can help visualize structures not commonly seen in the transthoracic approach, which include atrial septum, thoracic aorta, and pulmonary veins. Three-dimensional TTE has been found to be effective in calculating the mitral valve area (MVA) in patients with rheumatic mitral valve stenosis and has a great agreement with invasive methods [21]. Speckle tracking can detect sub-clinical cardiac involvement in RA, sometimes missed by conventional Doppler echocardiography [4]. On strain imaging, anakinra has shown improved left ventricular deformation [22]. Dobutamine stress echocardiography has detected silent myocardial ischemia in RA; it was mainly due to microvascular phenomena [23].

Myocardial perfusion imaging with SPECT enables the assessment of CAD noninvasively. It has been reported that myocardial perfusion imaging with PET has higher diagnostic accuracy than SPECT [24]. In nuclear studies for RA, dipyridamole thallium has shown microvasculitis and micro-thrombosis without evidence of MI or clinical symptoms [4]. Values of erythrocyte sedimentation rate (ESR) and rheumatoid factors such as IgM or IgG were found to be higher in RA with perfusion defects than normal perfusion RA [24]. The high costs associated with the technology have a limited role in these conditions [25] and require further studies.

Multi-slice CT with iodinated contrast agents enables visualization of the coronary lumen. It also identifies significant coronary artery stenosis and permits characterization of the atherosclerotic plaque. CT has shown very high accuracy in the early diagnosis of CAD [26]. The Agatson coronary calcium score reveals the magnitude of calcification present in the coronary arteries [27]. Mitral valve calcifications were independent predictors of early atherosclerosis in RA patients [28]. RA patients have an elevated occurrence of coronary plaques in the absence of CAD [2]. Residual disease activity was associated with an elevated risk of non-calcified and mixed plaques; this was linked to an augmented risk of potential cardiac events [29]. 

CT coronary angiography also permits the evaluation of the coronary arteries non-invasively. Compared to invasive coronary angiography, the sensitivity of both approaches is quite similar [30]. A normal CT angiography is considered very accurate in excluding CAD. CT coronary angiography can be used to rule out CAD in intermediate patients and to avoid invasive coronary angiography [30]. However, the radiation dose of CT coronary angiography can be higher. Nevertheless, there is still limited data about the role of CT angiography in rheumatoid arthritis [4].

CMR can non-invasively measure ejection fraction and volumes and provide three-dimensional images [4]. CMR has found lower mean ventricular mass in RA patients in reference to normal patients (p<.0001), showing reduced myocardial mass rather than hypertrophy in RA heart failure [31]. CMR has been used as a tool to assess the effect of tocilizumab on left ventricular dysfunction in RA patients [32]. CMR can detect ischemia in two different ways, which are either through dobutamine or a T1 shortening contrast agent. Perfusion CMR revealed more myocardial abnormalities in RA patients without known cardiac disease, suggesting inflammation plays a role in myocardial involvement [33]. CMR has also shown few instances of myocardial inflammation imitating myocarditis [34]. CMR is the most reliable modality to measure scar or fibrotic tissue [4].

Coronary magnetic resonance angiography (MRA) requires a lot of expertise and is gaining traction. It has been used in the evaluation of coronary arteries in RA and provided information about coronary lesions [4]. Further studies are needed.

Comparison of CMR with Other Modalities in Rheumatoid Arthritis

While echocardiography is the commonly used modality for assessing ventricular function, CMR has accurate measurements with good correlation [35]. Evaluation of the right ventricle is of considerable value in rheumatoid arthritis, which indicates the development of pulmonary artery hypertension and subclinical left heart failure. In some instances, this cannot be properly evaluated by echocardiography. CMR has more consistent and reproducible findings in relation to echocardiography [4]. Myocarditis is frequently undetected by nuclear imaging techniques or echocardiography in RA. The ability to characterize tissue such as myocardial inflammation makes CMR beneficial in these conditions. LGE has been used for the detection of myocardial necrosis in RA patients. In T2 weighted images and LGE in myocarditis, RA patients had increased end-diastolic volumes, decreased ejection fraction (P<.05), and diminished radial and longitudinal thickening (P< .01) [34]. The pattern of myocardial necrosis is mainly intra-myocardial or sub-epicardial similar to viral myocarditis which contrasts with ischemic heart disease which is sub-endocardial [4].

Systemic lupus erythematosus 

SLE is an autoimmune disorder with widespread organ involvement having a variable course [36]. It is typically characterized by remissions and exacerbations [36]. CVD is an important contributor to mortality [37]; MI frequently leads to heart failure [36]. 

Imaging Modalities in SLE

Echocardiography should be performed in all SLE patients irrespective of symptoms [36]. Left ventricular diastolic dysfunction is a common finding documented in echocardiography. As per the American College of Rheumatology (ACR), damage index > 1 had major left ventricular diastolic dysfunction with lower lateral annulus E’ [38]. For identifying left ventricular diastolic dysfunction, the E/e’ ratio is found to be more sensitive than the E/A ratio [39]. Tissue Doppler imaging can provide information regarding myocardial performance and subclinical heart disease better than conventional echocardiography [40]. Strain rate and strain imaging can depict systolic and diastolic left ventricular longitudinal function without cardiac symptoms [36].

Nuclear studies are frequently used to evaluate the level of myocardial ischemia in SLE patients prior to and following interventional procedures [36]. Perfusion abnormalities were recognized in symptomatic and asymptomatic SLE patients in SPECT [41]. In patients with low risk for CAD, SPECT identified CAD in low-risk asymptomatic patients [42]. Abnormal coronary blood flow has been observed in asymptomatic SLE patients in 13 ammonia PET. In symptomatic SLE patients with 18F fluoro-2-deoxy-D-glucose uptake on PET, abnormal glucose metabolism was seen with normal perfusion [43].

CT can evaluate calcification in the coronary artery to assess the extent of atherosclerotic progression [44]. Few studies have explored the role of CT in the evaluation of cardiovascular disease in SLE. Elevated levels of coronary artery calcifications in SLE have been linked to disease duration and activity [45]. In addition, CT in SLE patients has shown higher arterial and valvular calcification in reference to normal control subjects [46]. In myocarditis, delayed iodine contrast-enhanced CT has shown elevated uptake of contrast in the inflamed regions [47]. 

CMR is the imaging approach of choice for evaluation of the pathophysiologic process of heart disease in SLE and does not have concerns for radiation [39]. It can be the next step in diagnostic evaluation for SLE patients with persistent symptoms in the presence of conflicting results from routine testing [36]. CMR can reliably evaluate inflammation, fibrosis, perfusion, and function in SLE [39]. In SLE with heart failure, CMR can delineate various imaging patterns with significance [48]. Subclinical edema has been found in asymptomatic SLE patients by CMR [49]. In patients with a normal routine non-invasive assessment with cardiac symptoms, CMR has revealed myocarditis in children and myocardial infarction in adults [50]. 

Cardiac catheterization is an interventional approach that provides valuable diagnostic information in CAD. The information provided by cardiac catheterization can guide therapeutic intervention. For evaluating pulmonary artery pressures, cardiac catheterization can be hailed as the gold standard [36]. Limited studies have explored the role of cardiac catheterization in SLE. In one study with x-ray coronary angiography, SLE patients had a comparable severity of CAD with control patients [51]. The SLE patients were younger and many did not have diabetes mellitus. 

Comparison of CMR with Other Modalities in SLE 

Echocardiography is the most frequently used diagnostic modality for evaluating cardiovascular disease in SLE (36). As stated earlier, it is operator-dependent and can have poor acoustic windows in some patients. In patients with conflicting results during routine testing, CMR can provide additional information [36]. Cardiac catheterization is an invasive procedure and can be avoided or delayed by CMR in certain patients with atypical symptoms.

CT has been able to detect myocardial fibrosis in hypertrophic cardiomyopathy patients with contrast enhancement [52]. Studies have demonstrated excellent correlations between myocardial ECV by cardiac CT and T1 mapping [53]. There is a possibility that CT with contrast enhancement could be used for tissue characterization similar to CMR, and can be extended to SLE and other systemic diseases. However, the risk of radiation and the use of contrast agents limits its role in clinical care [1].

Systemic sclerosis

Systemic sclerosis (SSc) is characterized by abnormal collagen accumulation and fibrosis resulting in dysfunction of vascular organs. Cardiac involvement can occur secondary to fibrosis and PAH. Microvascular dysfunction and fibrosis can lead to compromised systolic and diastolic function [54].

*Imaging Modalities in Systemic Sclerosis* 

Early subclinical lesions can be seen with echocardiography, nuclear, and cardiac CMR studies [55]. These approaches can detect functional or fixed perfusion abnormalities [5]. T1 SPECT has been shown to identify a high incidence of abnormal findings in SSc patients [56]. In tissue Doppler imaging, various indices can be used to evaluate ventricular function [57]. One report has shown that tissue Doppler imaging was able to identify cardiac involvement and ventricular dysfunction in 100 patients with SSc; conventional echocardiography was not able to reveal any cardiac findings in the same patients [58]. Dobutamine stress echocardiography has been shown to detect microvascular defects in SSc [59]. CMR enables the cardiologist to obtain information regarding the myocardium, which can help monitor disease evolution and treatment response in SSc. CMR has recognized MI and diffuse or focal fibrosis even in the absence of cardiac symptoms [60]. Interestingly, adenosine stress CMR has identified asymptomatic perfusion defects earlier in SSc patients [61]. It has identified Q waves in myocardial fibrosis attributed to systemic disease [62]. T1 mapping and ECV quantification can evaluate more extensive forms of fibrosis [63]. T1 mapping and ECV can potentially be utilized for screening before overt left ventricular dysfunction occurs [60].

Comparison of CMR with Other Modalities in Systemic Sclerosis

The diagnosis and detection of myocardial fibrosis are not clearly defined in SSc [60]. Most noninvasive methods can detect ventricular abnormalities that are not associated with myocardial fibrosis [58]. Endomyocardial biopsy is the gold standard for diagnosis but is invasive in nature [64]. Echocardiography does not provide sufficient information on cardiac damage caused by fibrosis which can delay therapy [65]. CMR can provide information on tissue characterization and the results are reproducible. CMR can detect and quantify myocardial fibrosis. Furthermore, CMR can identify fibrosis better than echocardiography and nuclear approaches; it has an excellent correlation with findings on histology for animal and human studies [66]. 

Dobutamine stress echocardiography can identify wall motion abnormalities and provide information on myocardial blood flow [59]. Similarly, nuclear imaging approaches such as CT tomography and PET can detect irregularities in myocardial perfusion for SSc patients [67]. Cardiac catheterization cannot provide adequate information on cardiac involvement in SSc and is not frequently performed for these patients [68]. 

Sarcoidosis

Sarcoidosis is a granulomatous condition linked with damage to the myocardium [69-70]. There are a number of diagnostic issues in cardiac sarcoidosis due to the fundamental patchy nature of the condition and lack of a highly specific or sensitive test [8]. Though significant cardiac sarcoidosis can occur in close to 10% of patients, autopsies reveal close to 30% [71]. 

Role of Imaging Modalities in Sarcoidosis

The absence of a gold standard test and low sensitivity of the invasive endomyocardial biopsy has led to the diagnosis of sarcoidosis being made on clinical grounds [72]. An echocardiogram provides valuable insight if performed in conjunction with abnormalities in electrocardiograms or patients with cardiac symptoms [8]. Furthermore, an abnormal echocardiogram is very indicative of cardiac sarcoidosis [8]. A normal echocardiogram does not necessarily rule out sarcoidosis [8] and requires further testing. CMR can be advantageous because it provides greater detail and can be used in risk stratification [2]. In one report, CMR was found to be the superior test in diagnostic and prognostic capacity while echocardiography fared less in comparison [1].

Over many years, CMR and PET have been able to document cardiac involvement comparable to autopsy reports [73]. The Heart Rhythm Society recommends CMR and PET as diagnostic tools in the evaluation of cardiac sarcoidosis [74]. 18FDG PET can help distinguish between normal and active inflammatory lesions by showing increased glucose uptake and metabolic rate by macrophages [75]. A number of patterns can be seen in 18FDG PET, which includes diffuse, focal, and focal on diffuse [74]. The focal diffuse pattern is most typically associated with cardiac sarcoidosis with or without resting perfusion defects [76]. One report has shown abnormal 18 FDG uptake and a perfusion defect was linked to death and sustained ventricular tachycardia in cardiac sarcoidosis patients [77]. Another study has shown symptomatic cardiac sarcoidosis patients to have higher 18FDG uptake than asymptomatic patients [78].

CMR is frequently utilized in the assessment of cardiac sarcoidosis and can detect myocardial damage and fibrosis [1]. Once center found LGE to be a significant predictor of adverse events in sarcoidosis patients with non-specific symptoms [79]. T1 and T2 mapping can play a critical role in the early stages of sarcoidosis for recognizing cardiac involvement [80]. T2 mapping can be used to monitor progress following immunosuppressive therapy in cardiac sarcoidosis [81]. 

Systemic vasculitides

Systemic vasculitides consist of a spectrum of disorders characterized by inflammation and necrosis of the inner vessel wall of blood vessels [82]. They are frequently associated with malignancy, infection, and autoimmune disorders [82]. They contribute to elevated mortality risk during the later years of disease progression [83]. Prominent disorders in this entity include giant cell arteritis, Takayasu arteritis, Polyarteritis nodosa, Kawasaki disease, and Wegner’s granulomatosis.

Role of Imaging in Systemic Vasculitides

Though endomyocardial biopsy may be the gold standard in diagnosis, it is not always ideal due to the haphazard distribution of inflammation [82]. CMR enables tremendous versatility and greatly augmented spatial resolution, which can identify CVD manifestations in these conditions [82]. In addition to LGE, CMR may show elevated T1, ECV, and T2 values, which may provide additional information to supplement myocardial assessment in vasculitides [84]. CMR enables the detection of Takayasu arteritis at an earlier reversible stage and can evaluate disease response to treatment non-invasively [85]. In Giant cell arteritis, CMR can detect myocarditis and prompt immunosuppressive therapy to prevent left ventricular dysfunction [86]. In Kawasaki disease, CMR can show evidence of MI, left ventricular dysfunction, and microvascular disease, which can prompt management during acute phases [82] and great potential [87]. For risk stratification in treatment for anti-neutrophilic cytoplasmic antibody (ANCA) associated vasculitides for sustained remission, it has been suggested that CMR should be performed for all cases even for absent symptoms or normal ECG [88].

Mixed connective tissue diseases and myopathies

There are limited studies evaluating the potential of imaging for cardiac involvement in mixed connective tissue diseases. CMR can detect inflammation and fibrosis, which can possibly initiate treatment in mixed connective tissue diseases [1-89]. In contrast, CMR has been shown to reveal injury to the myocardium in patients with inflammatory myopathies without clinical features of cardiac involvement [90].

## Conclusions

The spectrum of cardiovascular features in systemic diseases is the result of various interactions among various pathological factors, which require multi-center studies and clinical trials to fully understand the multi-faceted nature of cardiac involvement in these complex heterogeneous conditions. The application of various imaging modalities can have a major diagnostic and prognostic impact on patient management. Even though there is no single imaging technique that has the high sensitivity, specificity, and reproducibility to detect inflammation, ischemia, scar, vasculitis, and microvascular dysfunction without limitations, CMR is assuring and superior among all the imaging techniques available.
